# Supplementary material for: A Diagnostic Gene Expression Signature for Bladder Cancer Can Stratify Cases into Prescribed Molecular Subtypes and Predict Outcome
Source: Diagnostics (Basel). 2022 Jul 25;12(8):1801. doi: 10.3390/diagnostics12081801 (PMC9332739; doi:10.3390/diagnostics12081801)
Supplement: Supplementary file 1 [file diagnostics-12-01801-s001.zip › Supplemental Figure S1.pdf]

Oncuria™

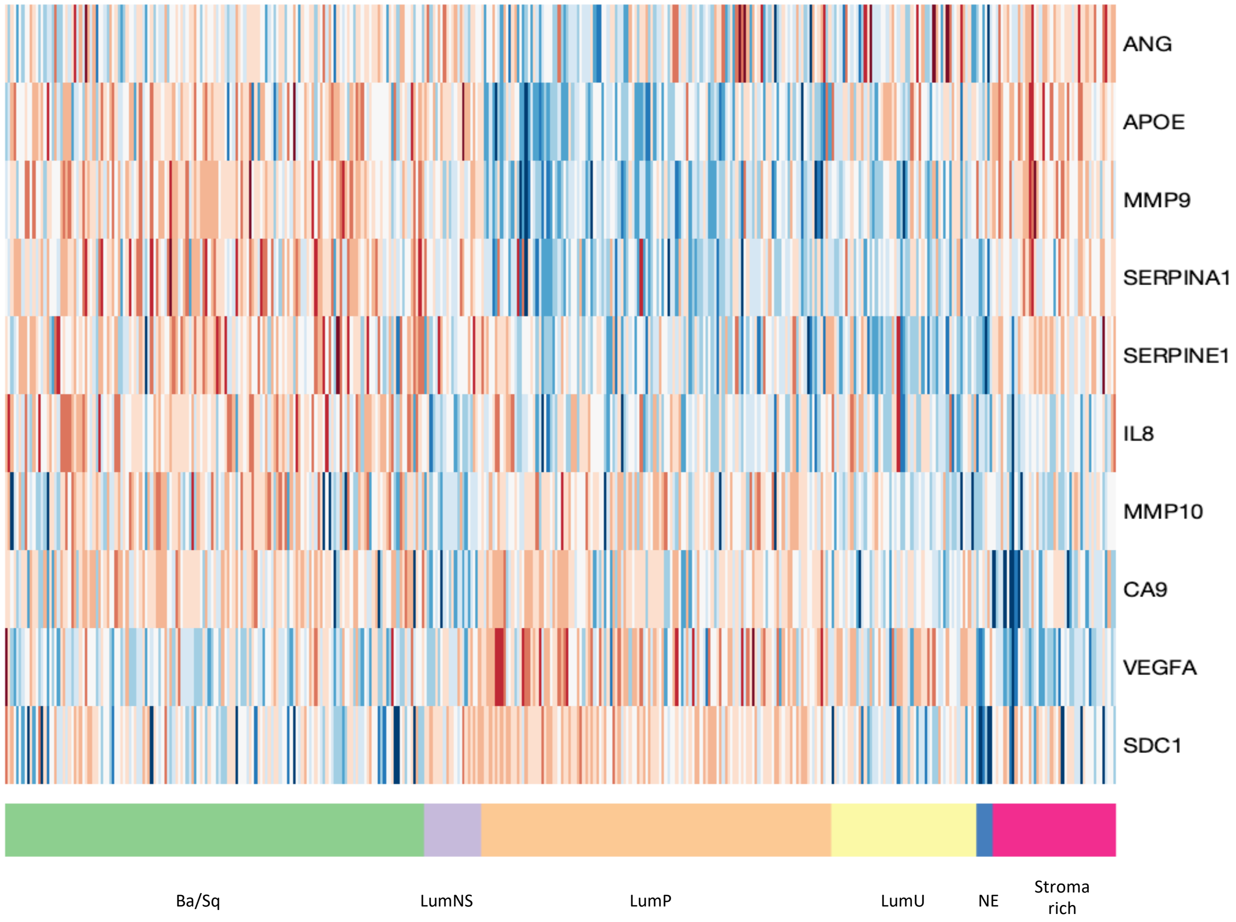

Supplemental Figure S1. Heatmap illustrating application of the 10 biomarkers of Oncuria™ associated with the 6 consensus molecular subtype in the TCGA cohort. Blue to Brown shows a trend from low to high gene expression.
