# Supplementary material for: A Diagnostic Gene Expression Signature for Bladder Cancer Can Stratify Cases into Prescribed Molecular Subtypes and Predict Outcome
Source: Diagnostics (Basel). 2022 Jul 25;12(8):1801. doi: 10.3390/diagnostics12081801 (PMC9332739; doi:10.3390/diagnostics12081801)
Supplement: Supplementary file 1 [file diagnostics-12-01801-s001.zip › Supplemental Figure S2.pdf]

Supplemental Figure S2. Kaplan–Meier survival curves for each of the 10 biomarkers associated with Oncuria™ in A) GSE87304, B) GSE48075 and C) GSE32894.

A) GSE87304

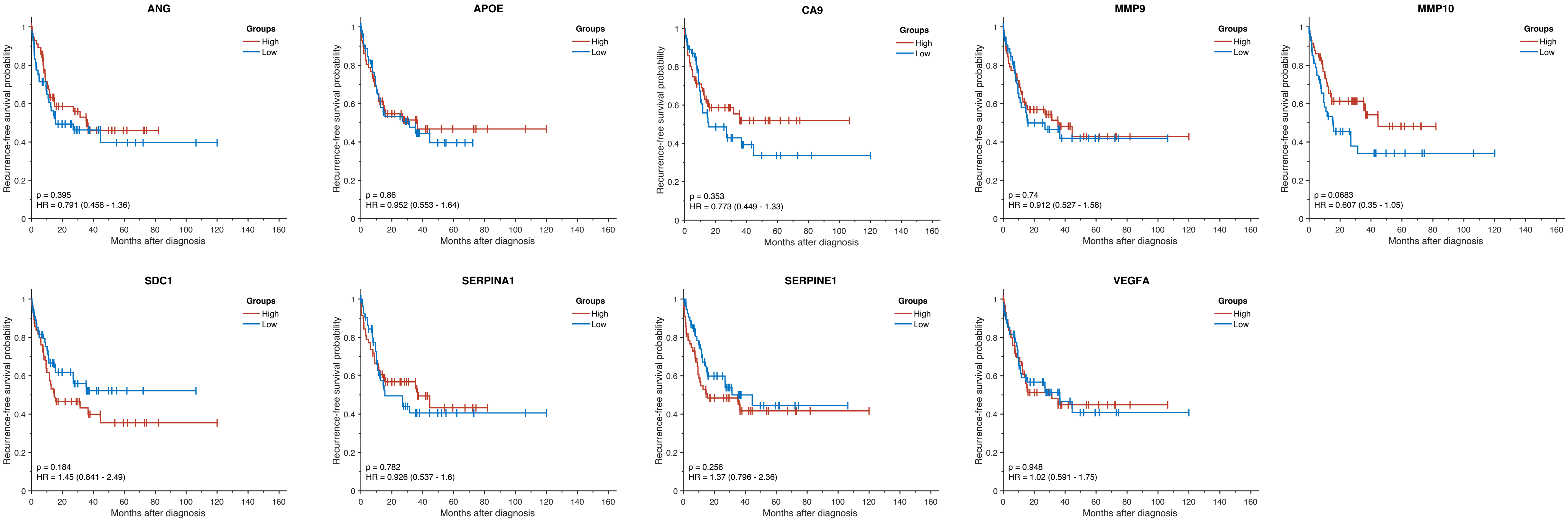

B) GSE48075

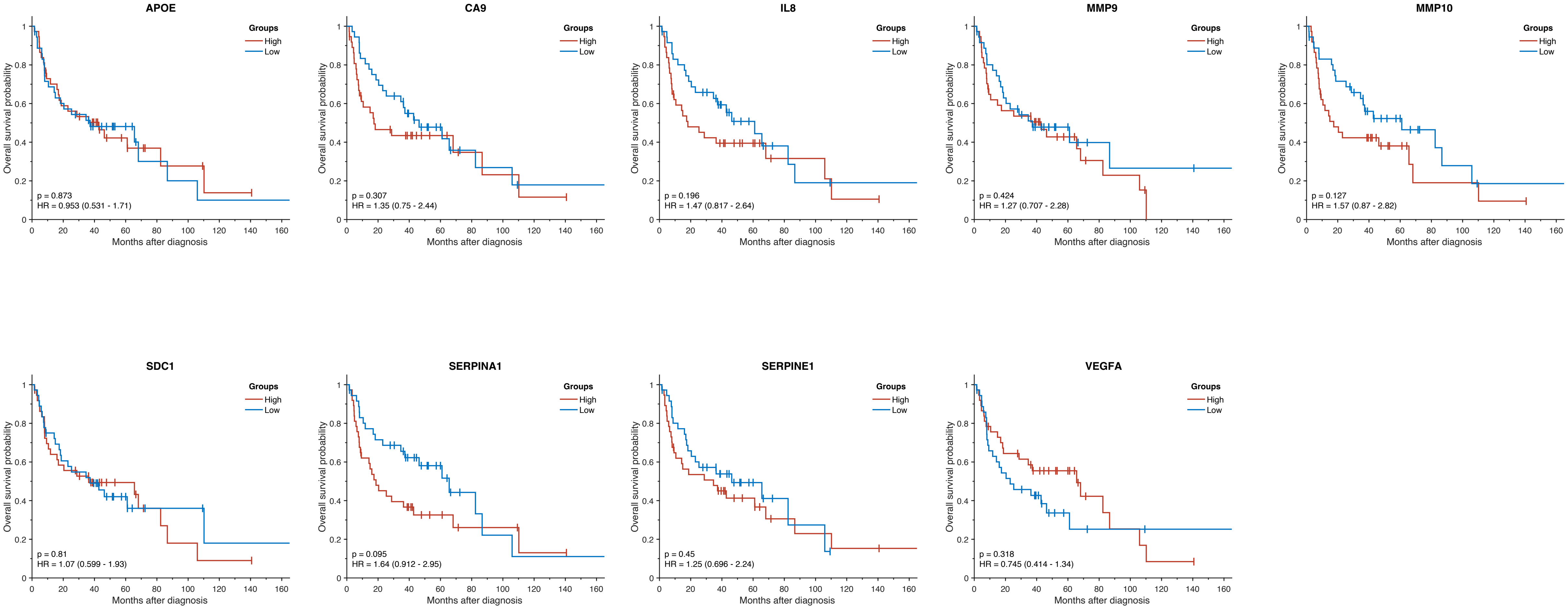
APOE
Groups
High
Low
Overall survival probability
Months after diagnosis
p = 0.873
HR = 0.953 (0.531 - 1.71)
CA9
p = 0.307
HR = 1.35 (0.75 - 2.44)
IL8
p = 0.196
HR = 1.47 (0.817 - 2.64)
MMP9
p = 0.424
HR = 1.27 (0.707 - 2.28)
MMP10
p = 0.127
HR = 1.57 (0.87 - 2.82)
SDC1
p = 0.81
HR = 1.07 (0.599 - 1.93)
SERPINA1
p = 0.095
HR = 1.64 (0.912 - 2.95)
SERPINE1
p = 0.45
HR = 1.25 (0.696 - 2.24)
VEGFA
p = 0.318
HR = 0.745 (0.414 - 1.34)

C) GSE32894

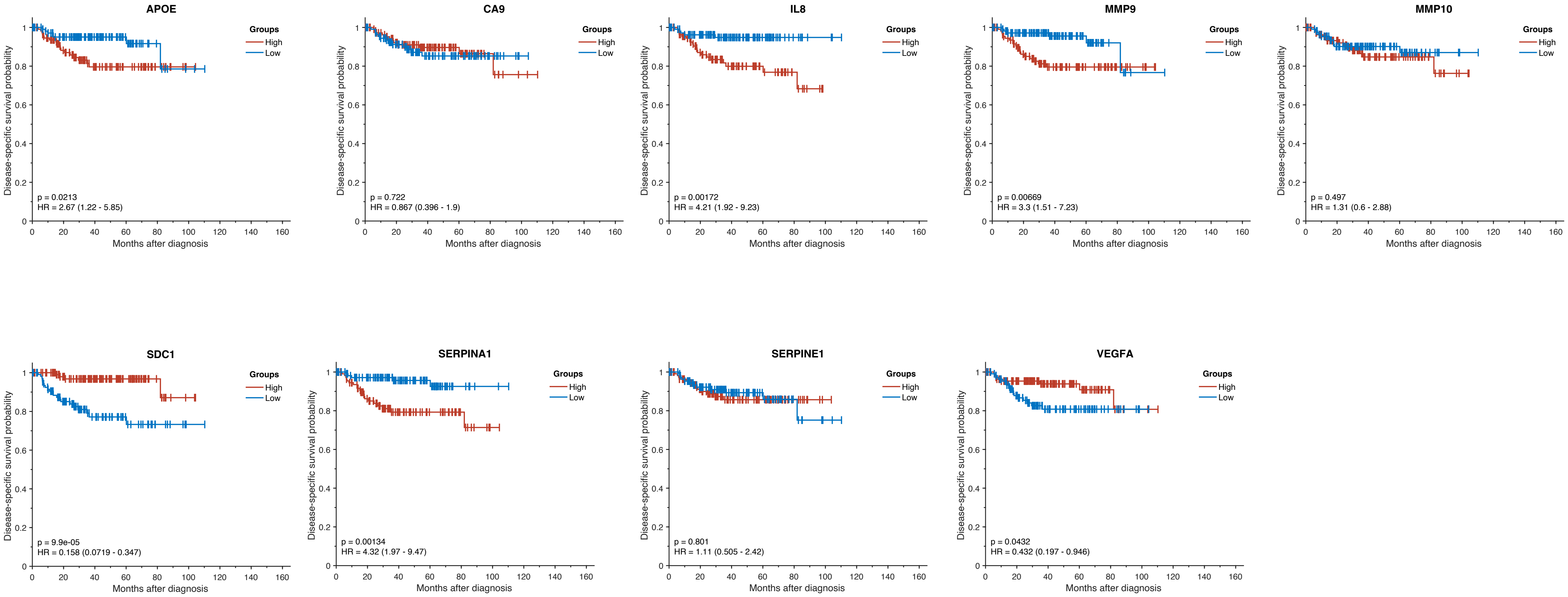
APOE
Groups
High
Low
p = 0.0213
HR = 2.67 (1.22 - 5.85)
CA9
p = 0.722
HR = 0.867 (0.396 - 1.9)
IL8
p = 0.00172
HR = 4.21 (1.92 - 9.23)
MMP9
p = 0.00669
HR = 3.3 (1.51 - 7.23)
MMP10
p = 0.497
HR = 1.31 (0.6 - 2.88)
SDC1
p = 9.9e-05
HR = 0.158 (0.0719 - 0.347)
SERPINA1
p = 0.00134
HR = 4.32 (1.97 - 9.47)
SERPINE1
p = 0.801
HR = 1.11 (0.505 - 2.42)
VEGFA
p = 0.0432
HR = 0.432 (0.197 - 0.946)
Disease-specific survival probability
Months after diagnosis
