# Supplementary material for: A Diagnostic Gene Expression Signature for Bladder Cancer Can Stratify Cases into Prescribed Molecular Subtypes and Predict Outcome
Source: Diagnostics (Basel). 2022 Jul 25;12(8):1801. doi: 10.3390/diagnostics12081801 (PMC9332739; doi:10.3390/diagnostics12081801)
Supplement: Supplementary file 1 [file diagnostics-12-01801-s001.zip › Supplemental Table S1.pdf]

Supplemental Table S1 Top biological processes and cellular component reported in the gene ontology pathway analysis of differentially expressed genes associated with a bladder cancer associated signature

	**Biological Process (Gene Ontology)**			
GO-term	Description	Count in network	Strength	False discovery rate
GO:0050930	Induction of positive chemotaxis	2 of 15	2.42	0.0301
GO:0043117	Positive regulation of vascular permeability	2 of 16	2.39	0.0304
GO:0043116	Negative regulation of vascular permeability	2 of 18	2.34	0.0304
GO:0030225	Macrophage differentiation	2 of 26	2.18	0.0385
GO:0002690	Positive regulation of leukocyte chemotaxis	3 of 98	1.78	0.0210
	**Cellular Component (Gene Ontology)**			
GO-term	Description	Count in network	Strength	False discovery rate
GO:0005615	Extracellular space	9 of 3195	0.74	0.00087
